# Effect of Thermomechanical Treatment of Al-Zn-Mg-Cu with Minor Amount of Sc and Zr on the Mechanical Properties

**DOI:** 10.3390/ma15020589

**Published:** 2022-01-13

**Authors:** Azam Beigi Kheradmand, Shamseddin Mirdamadi, Zahra Lalegani, Bejan Hamawandi

**Affiliations:** 1 Department of Mechanical Engineering, Shahrekord Branch, Islamic Azad University, Shahrekord 88137-33395, Iran; 2 Department of Materials Engineering, Science and Research Branch, Islamic Azad University, Tehran 82448-65179, Iran; mirdamadi@iust.ac.ir; 3 Young Researchers and Elite Club, Shahrekord Branch, Islamic Azad University, Shahrekord 88137-33395, Iran; z.lalegani@ut.ac.ir; 4 Department of Applied Physics, KTH Royal Institute of Technology, SE-10691 Stockholm, Sweden

**Keywords:** scandium, thermomechanical treatment, microstructure, 7000 series alloy

## Abstract

In this study, the mechanical and microstructural properties of Al-Zn-Mg-Cu-Zr cast alloy with 0.1% Sc under homogeneous, dissolution, and T6 and thermomechanical treatments with the aim of increasing the volume fraction of MgZn_2_. Al_3_(Sc,Zr) reinforcing precipitates were examined by hardness, microscopic examinations, tensile tests and software analysis. The results showed that, firstly, the hardness results are well proportional to the results of the tensile properties of alloys and, secondly, the strength of the alloy with thermomechanical treatments compared to T6 treatments increased from 492 MPa to 620 MPa and the elongation increased from 8% to 17% and was 100% upgraded. Microstructural and fracture cross section investigations showed that Al_3_(Sc,Zr) nanosize dispersoids were evenly distributed among MgZn_2_ dispersoids and the alloy fracture was of semi-ductile type and nanosize dispersoids less than 10 nm were observed at the end of the dimples in the fracture section. The volume fraction of nanosize dispersoids in the whole microstructure of thermomechanical treatment samples was also much higher than that of T6 heat treated samples, so that the percentage of Al_3_(Sc,Zr) precipitates arrived from less than 1% in T6 operation to 8.28% in the quench-controlled thermomechanical operation (with 50% deformation). The quality index (QI) in thermomechanical treatment samples is 19% higher than T6 samples, so that this index has increased from 641 in T6 operation to 760 in samples under thermomechanical treatment due to precipitate morphology, volume fraction of precipitates, their uniform distribution in the matrix, and nano sized precipitates in samples under thermomechanical treatment.

## 1. Introduction

Aluminum (Al) alloys are widely used in various industries. The addition of rare earth elements to Al alloys has been proven to have optimal effects [1,2,3,4,5,6,7]. Among Al alloy elements, scandium (Sc) is one of the most effective elements for Al reinforcement. Al-Sc alloys have excellent mechanical properties at ambient temperature and at high temperatures due to the presence of hard and densely structured Al_3_Sc particles [8,9,10]. Al_3_Sc precipitates remain coherent up to high temperatures with the Al matrix [11]. It has been shown that Al_3_Sc nanoparticles are formed in ageing or thermomechanical treatments [12,13]. Thermomechanical treatment is a metallurgical process contains the combination of a plastic or mechanical deformation process such as rolling, forging, or pressurized processes and a thermal process such as heat treatment, quenching in water, heating and cooling at different speeds in a single process [14,15,16]. The microstructure (precipitates or grain structure) and general properties of the alloys (mechanical properties or corrosion resistance) are affected by the heat and mechanical treatment performed on the alloy [17,18,19]. Among Al alloys, Al-Zn-Mg-Cu alloys along with Sc have utility in various industries. The 7000 series alloys (Al-Zn-Mg-Cu) are widely used in applications such as structural components, automotive and aerospace industries [20,21,22,23,24]. In recent years, researchers have studied the properties and microstructure of Al-Zn-Mg-Cu alloys under various treatments due to their high strength-to-weight ratio, relatively low price, good machinability, electrical conductivity, good corrosion resistance and excellent weldability [25,26,27,28,29,30]. Due to the high stacking fault energy of Al-Zn-Mg-Cu alloys, the hyper fine-grained structures are difficult to obtain by the conventional rolling process. For example, Al-Zn-Mg-Cu alloys treated by conventional thermomechanical processes have a grain size of more than 30 microns. Interestingly, secondary phase particles in Al-Zn-Mg-Cu alloys have been shown to play a role in grain modification during thermomechanical processing [31,32,33,34,35,36,37]. Therefore, the combination of alloying and the addition of elements, such as Sc, and plastic deformation and heat treatment to control these precipitates or particles during thermomechanical processing, can dramatically affect the microstructure evolution of Al-Zn-Mg-Cu alloys to modify grains and improve their properties. Adding elements such as Sc to this group of alloys and combining the resulting properties with the properties obtained from thermomechanical treatments creates Al_3_Sc particles. Al_3_Sc particles act as grain modifiers in the solidification process of Al alloys, reinforcing precipitates by fixing dislocations, and dispersed particles controlling the grain structure [9,38]. Considering the high price of Sc, it is better to add other alloying elements to the Al alloy along with this element, to improve the mechanical properties and reduce the cost. The combination of Sc and zirconium (Zr) leads to increased strength and reduced production costs of Al alloys. Because Zr is another effective and well-known alloying element that reduces the average grain size and improves the tensile strength by forming the Al_3_Zr particles [39].

The 7075 alloy is used in advanced industries [40,41], and the addition of Sc and Zr to this alloy improves its mechanical and microstructural properties [42]. In the present study, Sc and Zr were added to the 7075 series alloy and three-stage thermomechanical treatment was performed, and by examining the hardness, tensile strength, ductility, and QI index, these properties were compared with the properties obtained from T6 heat treatment. By performing the thermomechanical treatments mentioned in this study, precipitates of different sizes were created and were evenly distributed throughout the alloy matrix. This uniform distribution of precipitates in the alloy matrix caused a simultaneous increase in strength and elongation. To the best of our knowledge, no research has reported a simultaneous increase in strength and ductility of Al-Sc alloys.

## 2. Materials and Methods

### 2.1. Instrument

A resistive furnace with programmability and accuracy of ±3 degrees was used for heat treatment, and a Brinell hardness tester (AMsler, wolpert, Germany), according to the ASTM E10 standard [43], was used for hardness tests. A tensile test (Hounsfield instrument, H25KS model, England) was performed according to the ASTME8 standard [44], using small samples. Microstructure analysis was performed using field emission scanning electron microscopy (FESEM, MIRA3TESCAN-XMU, Australia) equipped with the second generation EDS microanalysis and high-resolution transmission electron microscopy (HRTEM, 200 kV Schottky field emitter, Zeiss 200 FE, Koln, Germany), and tensile properties analysis was performed using the parameters of tensile strength, yield strength and elongation.

In engineering applications, instead of using any of the parameters of tensile strength and elongation to failure separately, it is better to derive a more acceptable parameter from the combination of tensile strength and elongation. Therefore, in this study, the quality index (QI) was used [45,46,47]. QI obtains from the UTS semi-log plot in terms of elongation to failure from the following equation [48]:

QI = UTS(MPa) + 150 log(%El).(1)

Considering that the purpose of this study was to increase the volume fraction of precipitates and their uniform distribution using thermomechanical treatments, MIP software v4 was used to calculate the volume fraction of precipitates created in thermomechanical treatments and the results are compared with the volume fraction of precipitates from T6 treatment.

### 2.2. Preparation of Samples

Alloy samples were prepared by alloying and casting. For this purpose, alloy 7075 was first melted in a resistive furnace under argon gas and then Al-2Sc and Al-15Zr alloys were added to it and the initial Al_3_Sc and Al_3_Zr dispersoids were allowed to dissolve; then, before casting and for balancing the amount of zinc (Zn) and magnesium (Mg), Al-Zn and Al-Mg alloys were used and, finally, the melt was mixed with a graphite lancer and under argon gas. Then the casting was performed in a vertical cast iron mold. The amount of Sc and Zr in the alloy was analyzed by the ICP method and other elements were analyzed by quantometer analysis. Table 1 shows the chemical analysis of the alloy.

**Figure 1:**
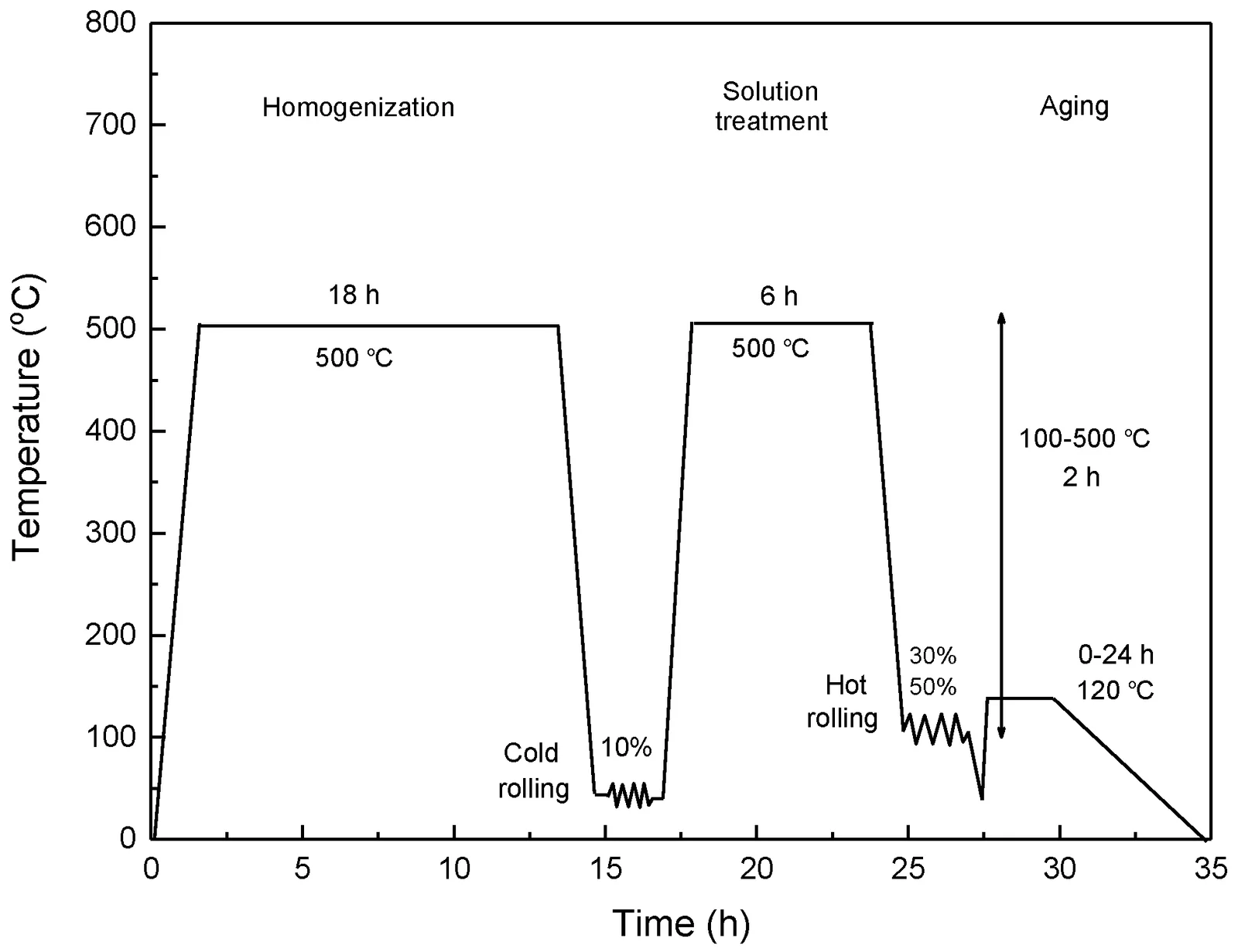
The schematic of thermomechanical treatments.

After alloying and casting, some specimens with the dimensions of 6 mm × 100 mm × 20 mm were cut from the center of the ingots. Then, the resulting specimens were machined and polished to remove contamination and surface scratches. Based on DSC analyses and metallographic studies, which are reported in another work by the authors [49], the temperature and time of the homogenization of specimens were obtained. The homogenization temperature and time for the alloy were 500 °C and 18 h, respectively. Homogenization is typically carried out at 450−500 °C, but temperatures can vary widely depending on the alloy composition [50]. The homogenization temperature in Sc-containing Al alloys also varies according to the chemical composition, so that in a study by Marquis and Seidman [51] for Al-Mg-Sc alloy, this temperature is mentioned as 618 °C, and in another study by Li et al. [52] for Al-Cu-Li-Sc-Zr alloy, this temperature is stated as 520 °C. After homogenizing the specimens, the thickness of the specimens was reduced from 6 mm to 4 mm by a hot rolling process at 500 °C. Secondary annealing was then performed for 6 h and the dissolution temperature of the specimens was obtained based on an electrical resistance test and a hardness test. Dissolution of specimens was performed in two ways: normal and controlled quenching. After dissolution, the thickness of the specimens decreased from 4 mm to 2.8 mm (30% rolling) and 2.0 mm (50% rolling). The cross-section reduction process was performed by rolling at 100 °C so that before each rolling pass, the specimens were kept at 100 °C for 3 min and were then rolled. After reaching the desired thickness, the specimens were exposed to 100, 120, 130, 140, 150, 200, 250, 300, 350, 400, 450, and 500 °C for 2 h to investigate the effect of temperature on hardness. To investigate the effect of time, the specimens were subjected to ageing treatments at a constant temperature of 120 °C for 10 min to 24 h. In order to obtain accuracy in hardness measurements, three specimens were tested at each temperature and time. The microstructure of thermomechanical specimens was investigated by metallographic, FESEM, EDS, and TEM analysis. Aged specimens were subjected to tensile testing (according to ASTM E8 standard) at the peak time and temperature of age hardening, and the mechanical properties of the specimens were obtained. The QI index was calculated and, finally, while investigating the fracture surfaces of the specimens with FESEM and EDS analysis, precipitate distribution and size of dimples were analyzed using MPI software. The specifications of the specimens and the performed thermomechanical treatments are presented in Figure 1 and Table 2.

## 3. Results

### 3.1. Effect of Ageing Temperature on Hardness

**Figure 2:**
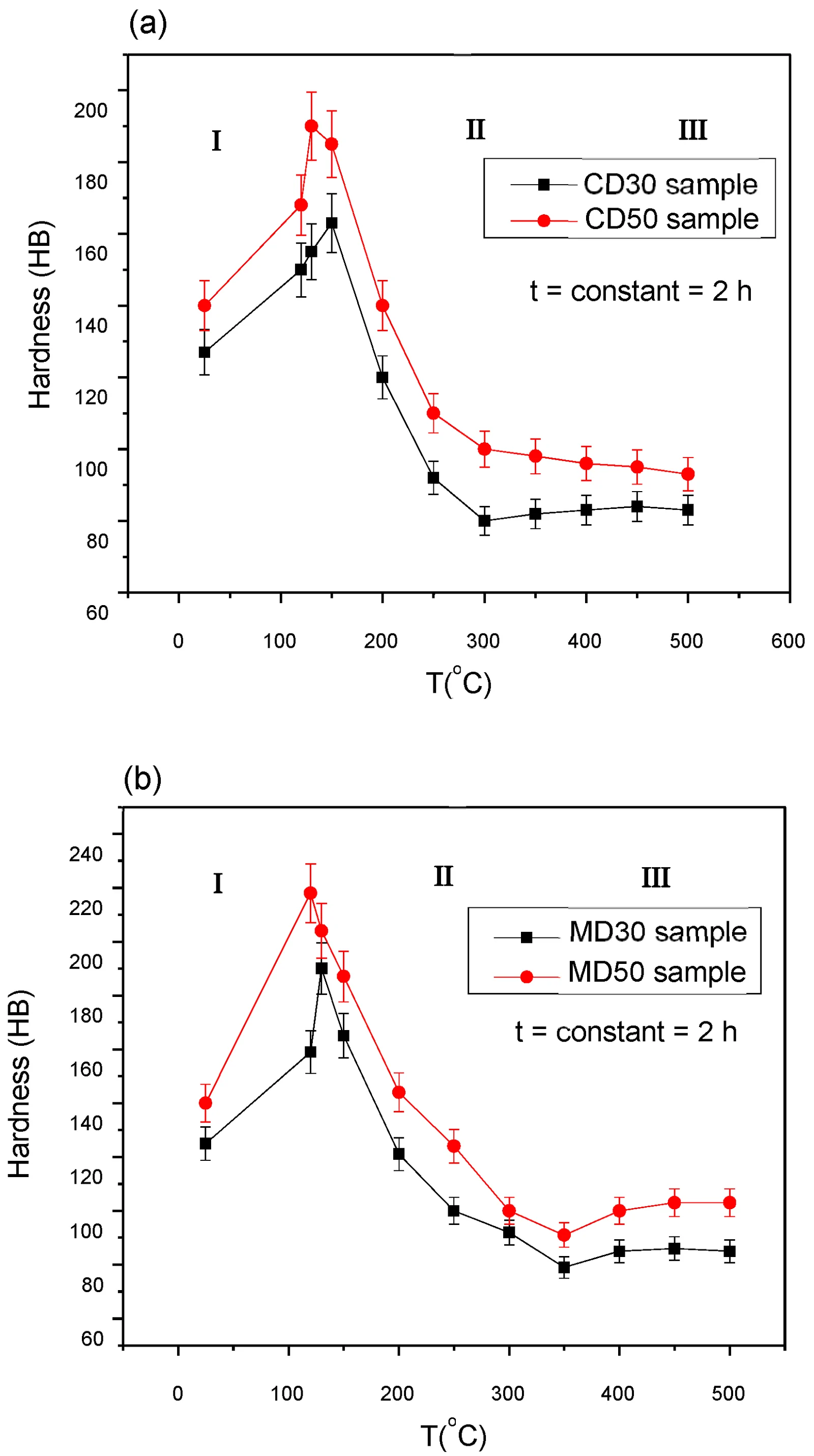
The effect of ageing temperature on the hardness of alloys in thermomechanical treatments with: (**a**) conventional dissolution, and (**b**) modified quenching.

Figure 2 shows the effect of ageing temperature on the hardness of alloys in thermomechanical treatments with modified quenching and conventional dissolution. As can be seen, the peak hardness temperature for alloys with 30% and 50% rolling are 130 °C and 120 °C, respectively. It is also observed that the hardness of alloy CD30 is higher than the hardness of alloy MD30. In fact, the hardness of specimens with controlled quenching thermomechanical treatment is higher than the hardness of specimens with conventional dissolution treatment.

According to Figure 2a, it can be seen that, in thermomechanical treatments with conventional dissolution, the hardness also increases with increasing the deformation rate. Comparison of the results of thermomechanical treatments with conventional dissolution and thermomechanical treatments with controlled quenching (Figure 2b) shows that the peak hardness in thermomechanical treatments with controlled quenching has increased compared to the peak hardness in thermomechanical treatments with conventional dissolution. So that these values for samples CD50 and CD30 are 195 and 160 Brinell, respectively, and for samples MD50 and MD30 they are 220 and 185 Brinell, respectively. On the other hand, it can be seen that in both treatments, the peak hardness temperature for the alloys with 50% and 30% rolling are 120 °C and 130 °C, respectively. In both curves (Figure 2a,b), three areas can be observed. The first zone is associated with hardness increasing. The second zone is the over ageing zone, where the density of growing precipitates appears to be greater than the density of nucleating precipitates. This growth of precipitates leads to a decrease in hardness. It may be said that in the aged state, on the one hand, the amount of solid solution is insignificant and, on the other hand, the precipitates in the matrix begin to thicken. This makes it easier for the dislocations to move, and thus reduces the hardness. In the third zone, the hardness is almost constant.

### 3.2. Effect of Ageing Time on Hardness

**Figure 3:**
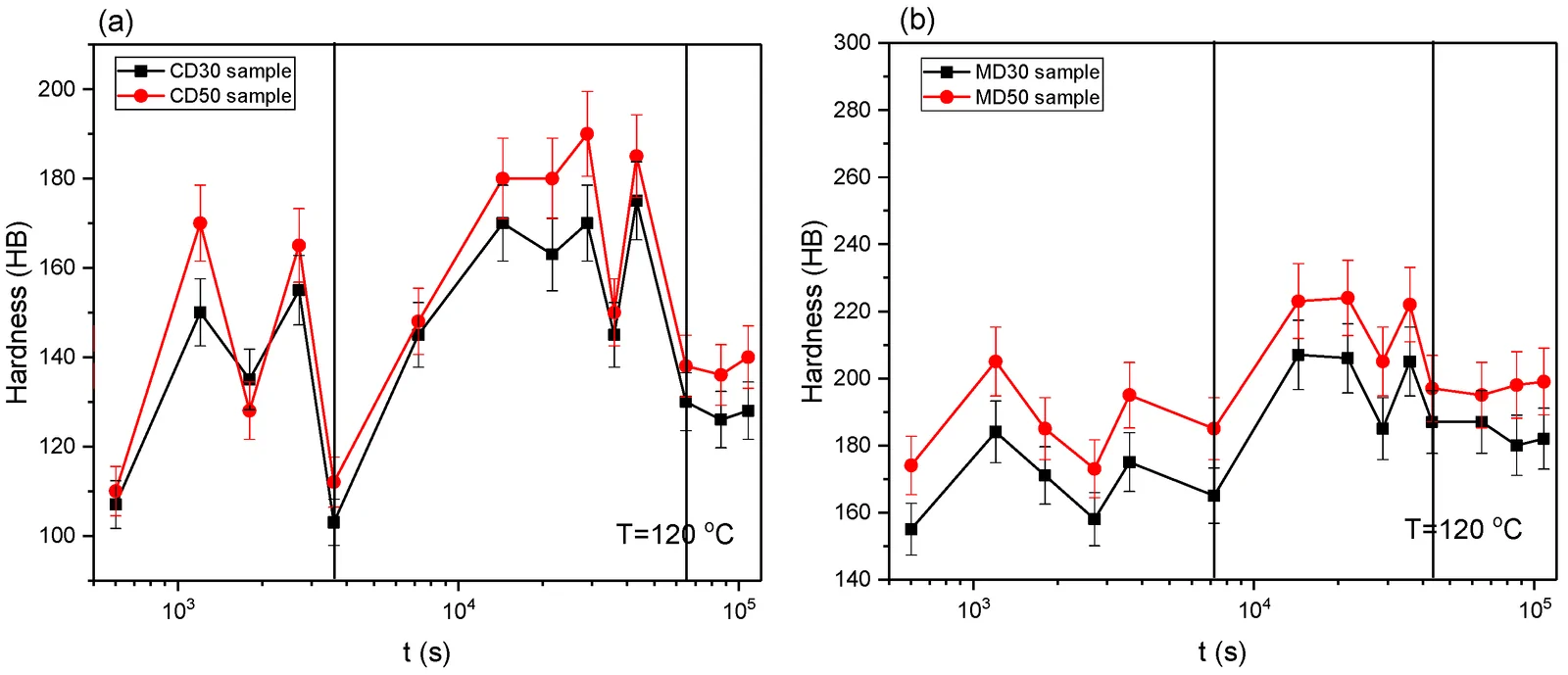
The effect of ageing time on the hardness of alloys in thermomechanical treatments with: (**a**) conventional dissolution, and (**b**) modified quenching.

Figure 3 shows the effect of ageing time on the hardness of specimens. As can be seen, both curves fluctuate with increasing time without a constant trend, but the maximum hardness in thermomechanical treatments with controlled quenching is higher than that with conventional dissolution. As mentioned, the hardness of specimens with controlled quenching thermomechanical treatments is higher than the hardness of specimens with conventional dissolution treatments. It can be related to the method of dissolution treatments and its effect on the number of precipitates. In controlled quenching, dissolution and quenching treatments increase the diffusion coefficient and activate energetic centers for precipitate nucleation. Therefore, in this case, the number of precipitate nucleation sites increases. On the other hand, thermomechanical treatments produce many high-energy centers that require the least energy for nucleation, which is a good source for precipitation, and the number of precipitates increases and their distribution becomes uniform.

According to Figure 3, it can be seen that, in the first hour of ageing, the hardness fluctuates due to the interaction of dislocations and precipitates. This fluctuation can be due to the effects of ageing and partial recrystallization. The hardness fluctuates until the effects of ageing predominate and the hardness of the alloy increases due to the increase in the number of precipitates. Henceforth, the hardness fluctuation will be low and eventually the hardness of the specimens will remain almost constant and the hardness of the specimens with 50% deformation will be more than the specimens with 30% deformation. It is also observed that at times of more than 16 h, the hardness is almost constant due to the thermally stable Al_3_(Sc,Zr) precipitates. According to DSC analyzes performed by Zhang et al. [53], the phenomenon of multiple peaks in the aged alloy at a temperature of 120 °C can be analyzed. According to Zhang’s analysis, the ageing process can be separated into three stages: the formation of small primary Guinier Preston (GP) zones; roughening of GP; and the precipitation of eta prime phase. In the early stages of precipitation, strong bonding of Zr and void may delay the nucleation of the eta prime phase and thus reduce the number of precipitates at this stage [54], which leads to a decrease in hardness. With ageing for more than 10 h, the number of GP zones increases and more eta prime phases are formed; also, the number of Al_3_(Sc,Zr) dispersoids increases in this region, therefore the second peak of ageing is created. In this region, the combined effect of Al_3_(Sc,Zr) and MgZn_2_ dispersoids leads to a further increase in hardness, so the second peak has a higher hardness. The Al_3_(Sc,Zr) phase is coherent with the Al matrix [44], and these coherent particles have very good thermal stability due to their low solubility and very low diffusion coefficient in the Al matrix [55].

Comparing the maximum hardness obtained in T6 hardening treatment with thermomechanical treatment, it is observed that the maximum hardness obtained in thermomechanical treatment is higher than the T6 hardening treatment. This increase in hardness is related to the deformation, which was performed before the ageing treatment. During deformation, the interaction of two sets of processes controls the behavior of the material: material softening processes including partial recovery and recrystallization and hardening processes including the increasing of dislocations density and the formation of precipitates. The main variables affecting these processes are the deformation temperature, the method of dissolution treatment, and the applied deformation rate. Under the same conditions of dissolution treatment, the deformation before ageing increases the diffusion coefficient; and as the deformation rate increases, the density of the dislocations increases. These dislocations are regions for heterogeneous nucleation as well as rapid diffusion pathways. Therefore, due to the increase in diffusion power, the high-energy centers for nucleation are increased and, by rolling at 100 °C, the effects of dislocations are recovered and, during ageing at 120 °C, the volume fraction of precipitates increases.

### 3.3. Microstructural Investigation

In metallographic studies, the size and shape of the grains (coaxial or elongated) are considered. The microstructure of the alloy CD50 is shown in Figure S1. Figure S1 shows elongated grains in the rolling direction and intermetallic particles.

The microstructure of the alloy containing 0.1% Sc after thermomechanical treatments with controlled quenching and different deformation is shown in Figure S2. In Figure S2a,c, elongated grains in the direction of rolling and black particles are seen at some grain boundaries that were insoluble during the dissolution treatment. In Figure S2b,d, few coaxial grains can be seen.

**Figure 4:**
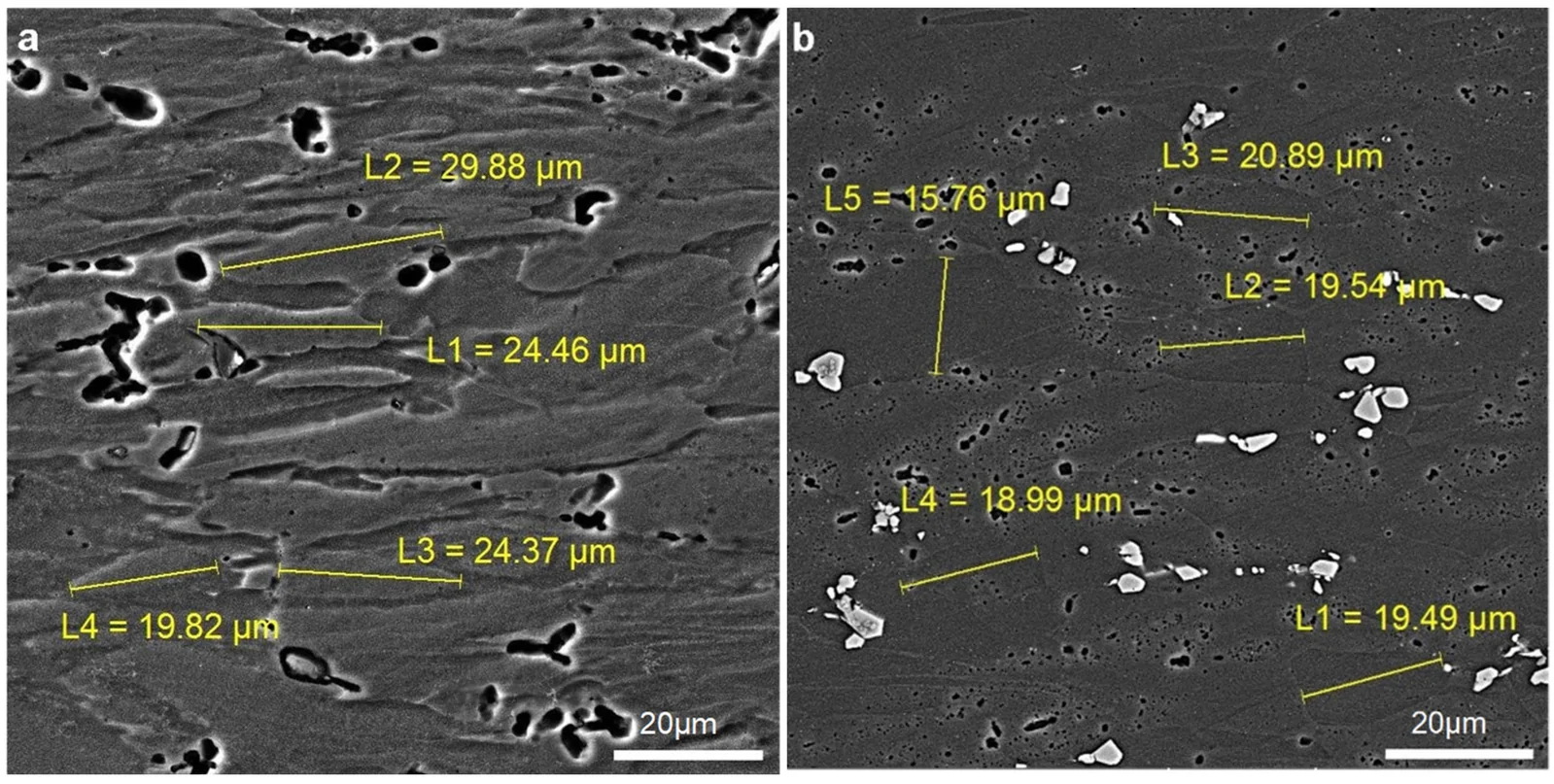
Grain size in alloy containing 0.1% Sc under two thermomechanical treatments with 50% deformation: (**a**) thermomechanical treatments with conventional dissolution, and (**b**) thermomechanical treatments with controlled quenching at 120 °C for 12 h.

In order to more precisely study the effect of thermomechanical treatments and also the effect of deformation rate and dissolution method on alloy microstructure, the alloy microstructure in 50% and 30% deformations and also in two methods of conventional dissolution and controlled quenching treatments were investigated. The purpose of this study was to determine the grain size and volume fraction of precipitates generated during the process. Figure 4 shows the grain size in the alloy CD50 and MD50. The grain size in FESEM images was measured by the linear intercept method [56,57]. The average grain length in Figure 4a is 25 μm and in Figure 4b is 19 μm. This is in accordance with the metallographic study of these specimens (Figures S1 and S2).

**Figure 5:**
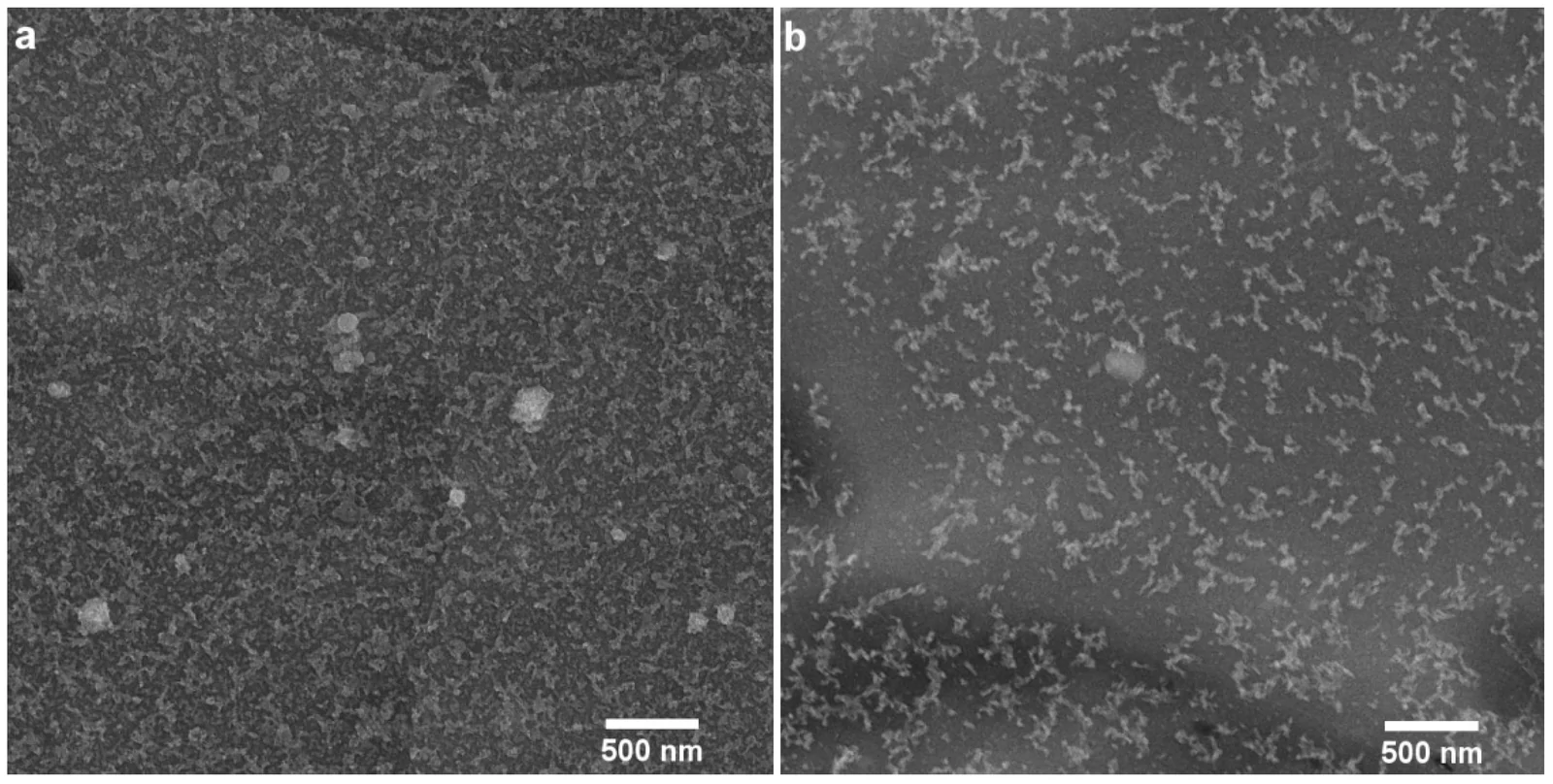
Precipitate distribution in two alloy specimens containing 0.1% Sc under thermomechanical treatment with 50% deformation at 120 °C for 12 h: (**a**) with controlled quenching, and (**b**) with conventional dissolution.

The distribution of precipitates in the samples CD50 and MD50 is shown in Figure 5. An FESEM (MIRA3TESCAN-XMU equipped with the second generation EDS microanalysis, Australia) has been used to analyze the precipitates. Figure 5a shows white spherical precipitates with an average size of 100 nm and Figure 5b shows white spherical precipitates with an average size of 186 nm. In both images (Figure 5a,b), two types of precipitates can be seen. There are also a number of disk-shaped precipitates that have been created by the accumulation of spherical precipitates together.

**Figure 6:**
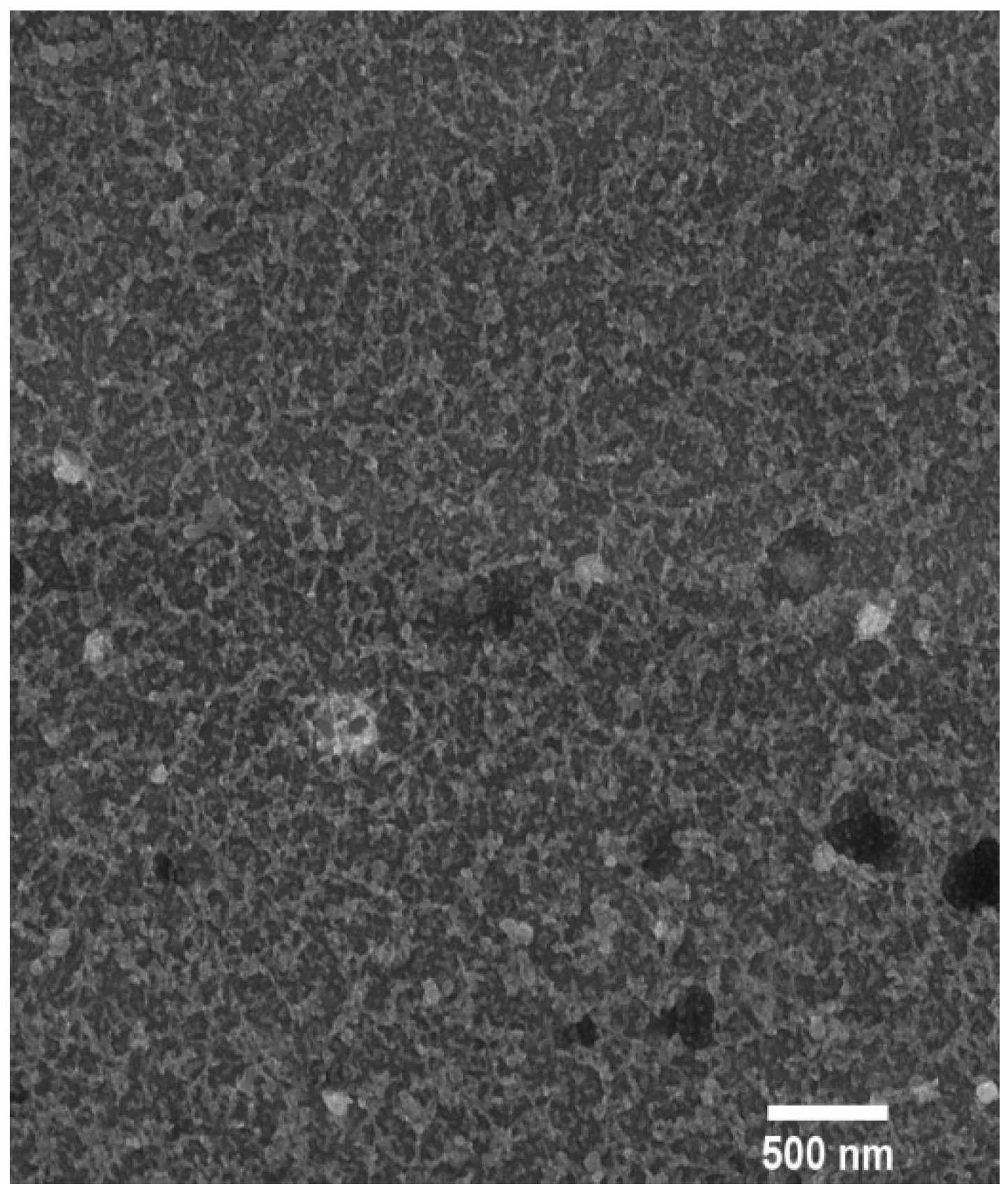
Distribution of precipitates in the sample CD30 and ageing at 120 °C for 12 h.

The distribution of precipitates in the sample CD30 is shown in Figure 6. In this figure, two types of precipitates can be seen; some of them are spherical and white, and most of them are light gray, which are distributed in the Al matrix. The average size of spherical precipitates is 90 nm.

**Figure 7:**
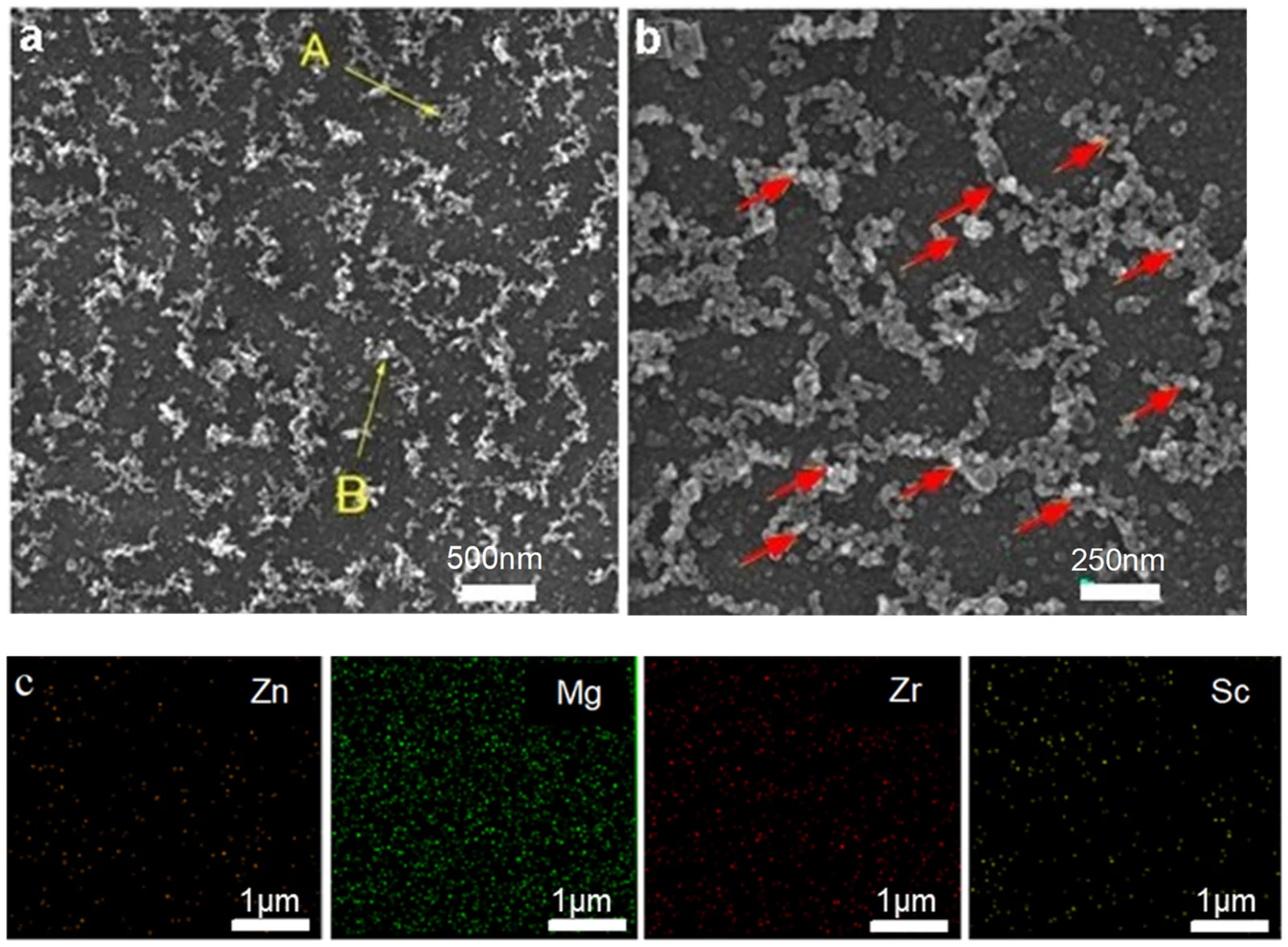
Distribution of MgZn_2_ and Al_3_(Sc,Zr) precipitates in Al matrix in alloy under thermomechanical treatment with conventional dissolution and 30% rolling: (**a**) Al_3_(Sc,Zr) and MgZn_2_ precipitates, (**b**) distribution of Al_3_(Sc,Zr), and (**c**) element distribution maps.

The distribution of MgZn_2_ and Al_3_(Sc,Zr) precipitates in the matrix in the sample CD30 along with the element distribution map are given in Figure 7. Figure 7a, taken at 50,000× magnification, shows two types of precipitate: type B precipitates are spherical and white, and type A precipitates are light gray, which are seen in the shape of a disk from the accumulation of precipitates together. In Figure 7b, type B precipitates are indicated by red arrows. Figure 7c shows the elements distribution map. As can be seen, Zn, Mg, Sc and Zr elements are distributed in the matrix. The distribution of Zn and Mg elements are observed in the whole microstructure, and the distribution of Sc and Zr elements are observed in some parts of the microstructures.

The elemental analysis of the phases is given in Table 3 (according to Figure 7).

According to this table, phase A is rich in Zn and Mg and the ratio of Zn to Mg is 2:1. Phase B is rich in Sc and Zr, and the composition of Sc and Zr is higher than the chemical composition of the primary alloy.

**Figure 8:**
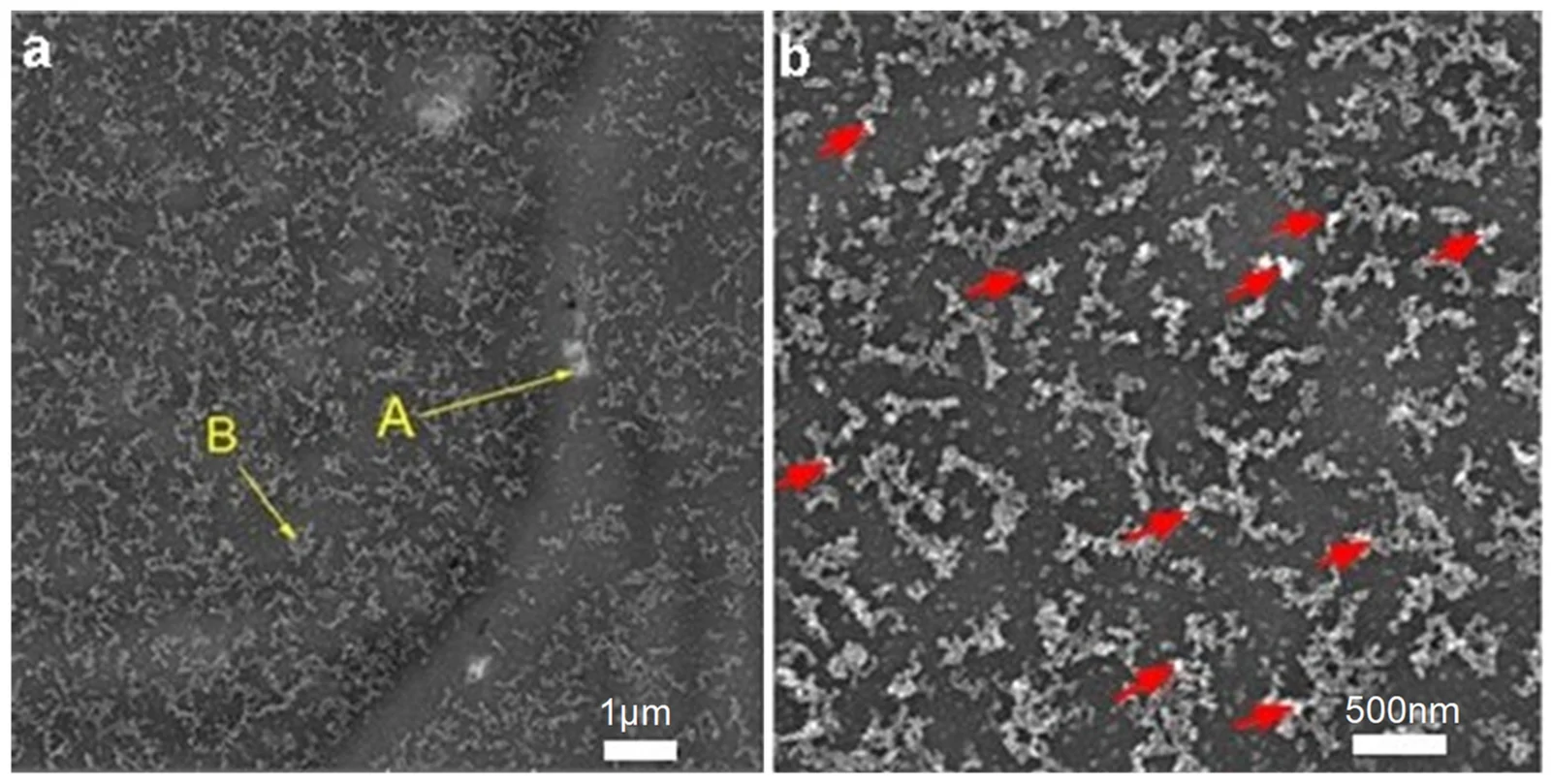

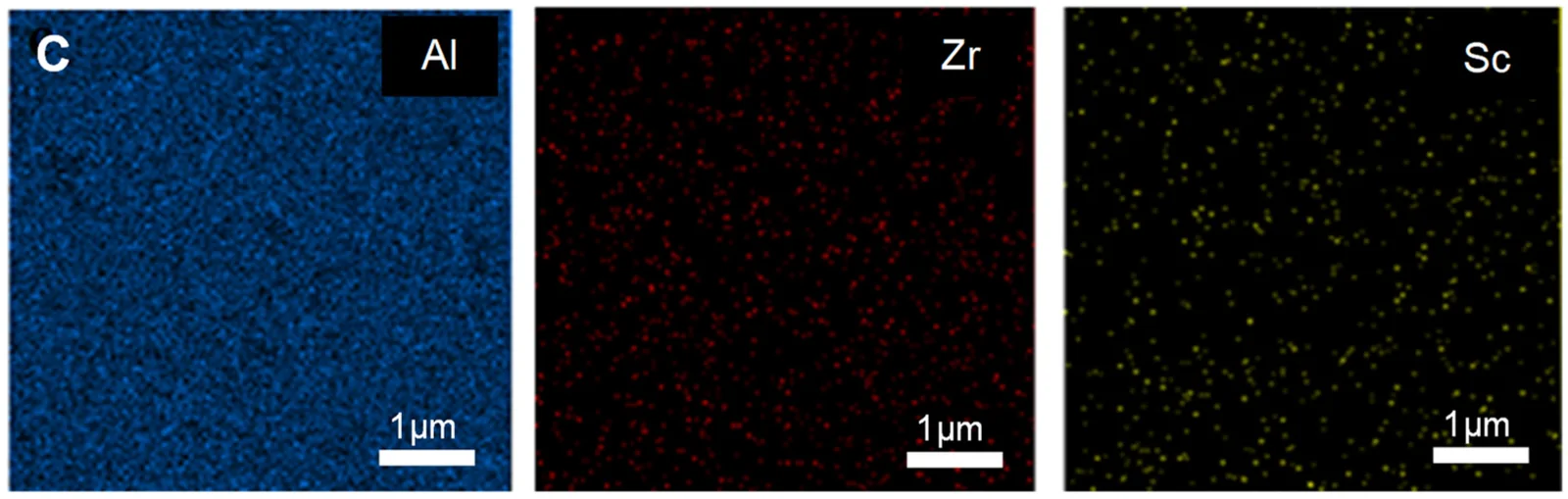
Distribution of MgZn_2_ and Al_3_(Sc,Zr) precipitates in Al matrix in the alloy containing 0.1% Sc, under thermomechanical treatment with controlled quenching and 30% rolling: (**a**,**b**) Distribution of MgZn_2_ and Al_3_(Sc,Zr) precipitates, and (**c**) element distribution maps.

The distribution of precipitates in the alloy MD30 is shown in Figure 8.

Figure 8a shows two types of precipitate, A and B, with different contrast. Precipitate A is found slightly at the grain boundaries and mostly inside the grain. Precipitates A are light white and precipitates B are light gray. Figure 8b shows the distribution of A precipitates and is shown with red arrows. EDS analysis of these phases is shown in Table 4. Figure 8c shows the distribution map of Sc and Zr elements in the microstructure. The densities of Sc and Zr are higher in some parts of the microstructure. According to Table 4, phase A is rich in Sc and Zr and the percentage of Sc and Zr elements in this phase is much higher than the initial chemical composition of the alloy. Phase B is also rich in Zn and Mg and the ratio of Zn to Mg is 2:1.

**Figure 9:**
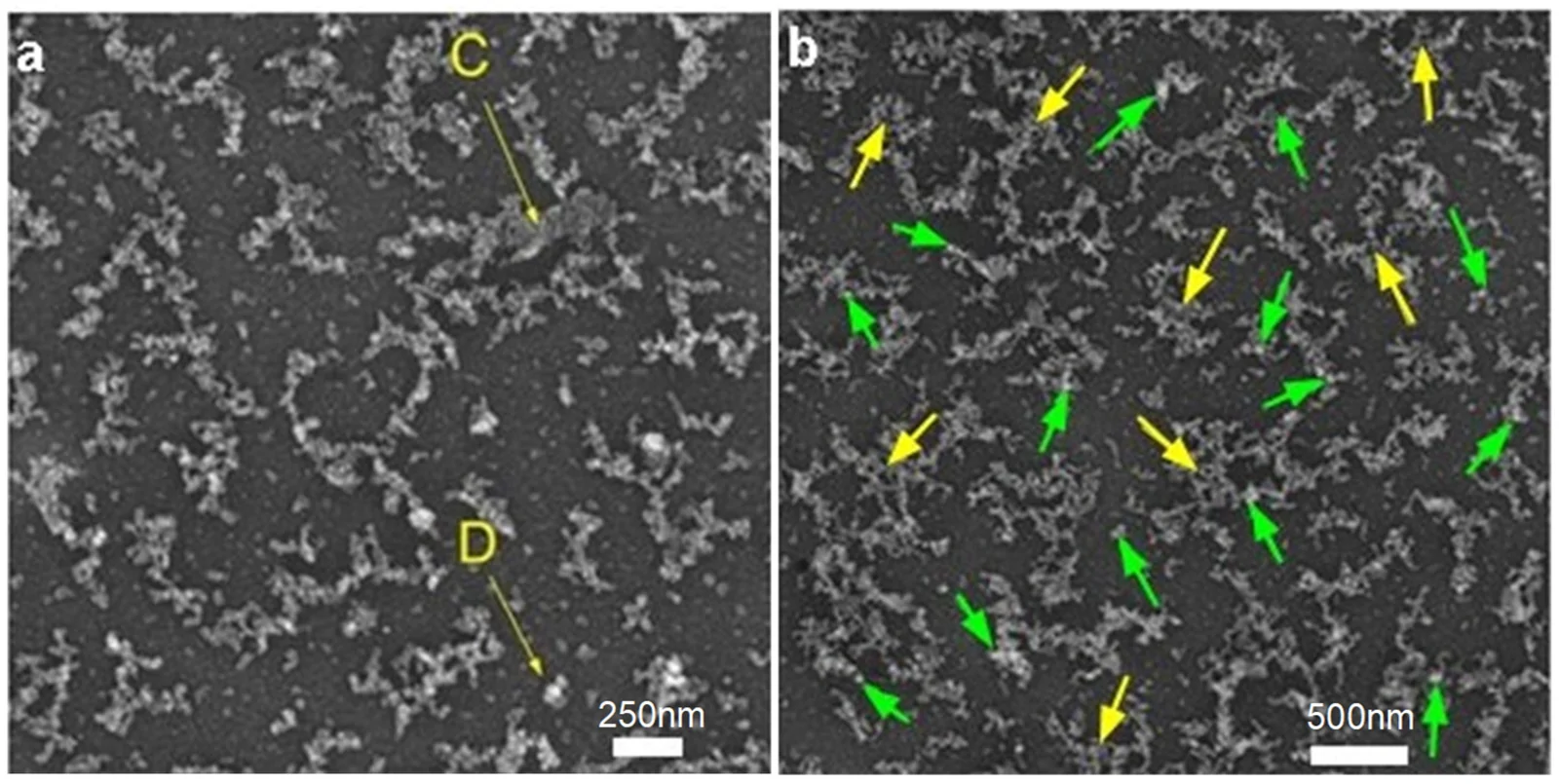

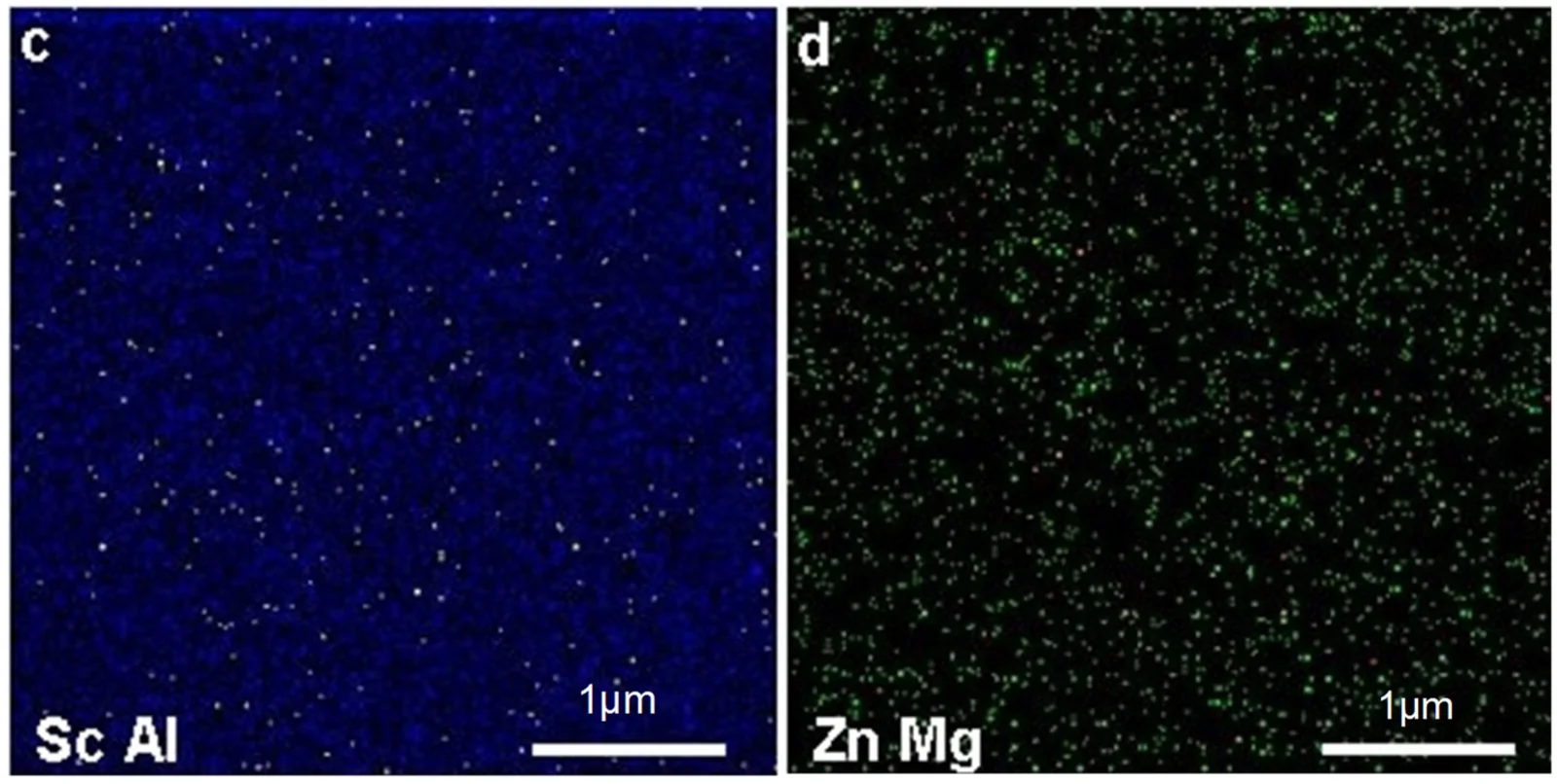
Distribution of MgZn_2_ and Al_3_(Sc,Zr) precipitates in Al matrix in the alloy containing 0.1% Sc, under thermomechanical treatment with controlled quenching and 50% rolling: (**a**,**b**) Distribution of MgZn_2_ and Al_3_(Sc,Zr) precipitates, (**c**) element distribution maps of Al-Sc elements, and (**d**) element distribution maps of Zn-Mg elements.

Precipitate distribution in the sample MD50 is shown in Figure 9. Figure 9a shows two types of precipitate with different contrasts. The type D precipitates are spherical and white and the type C precipitates are light gray. Figure 9b shows the distribution of type D precipitates with green arrows and the distribution of type C precipitates with yellow arrows. In Figure 9c, the element distribution maps of Al and Sc are shown and it is observed that the density of Sc element is higher in some parts of the matrix. Figure 9d shows the element distribution map of Zn and Mg and shows that they are distributed in the whole matrix.

The elemental analysis of the phases of Figure 9 is presented in Table 5. According to Table 5, C precipitates are rich in Mg and Zn and the ratio of Zn to Mg in this phase is 2:1. D precipitates are rich in Sc and Zr, and the amount of Sc and Zr in this phase is several times higher than that of Sc and Zr in the chemical composition of the primary alloy.

According to FESEM images and EDS analysis of precipitates, Al_3_(Sc,Zr) and MgZn_2_ precipitates are observed; these precipitates are uniformly distributed in the microstructure. Tao and Romestch have also shown that Al_3_(Sc,Zr) dispersoids are formed in thermomechanical treatments [58,59]. Total microscopic observations on the distribution of precipitates in samples MD50 and CD50 at 100 °C show that the number of Al_3_(Sc,Zr) precipitates in the specimen under thermomechanical operation with controlled quenching is more than the specimen undergoing thermomechanical treatment with conventional heat treatment. It seems that increasing the diffusion rate due to controlled quenching increases the nucleation sites of the precipitate and thus increases the number of precipitates.

It is also observed that, under the same ageing conditions (50% rolling at 100 °C and ageing at 120 °C for 12 h), the grain size in the alloy under thermomechanical treatments with controlled quenching is less than the grain size in the alloy under thermomechanical treatments with conventional dissolution. The presence of more Al_3_(Sc,Zr) dispersoids in the alloy and their inhibitory effect on grain growth due to controlled quenching, have optimized the grain size. Since the number of precipitates in the alloy under controlled quenching thermomechanical treatments is higher than the alloy under conventional dissolution thermomechanical treatments, then the grain structure is modified. Minor amounts of Sc and Zr improve the grain structure and alloy strength and restrict the grain [60].

**Figure 10:**
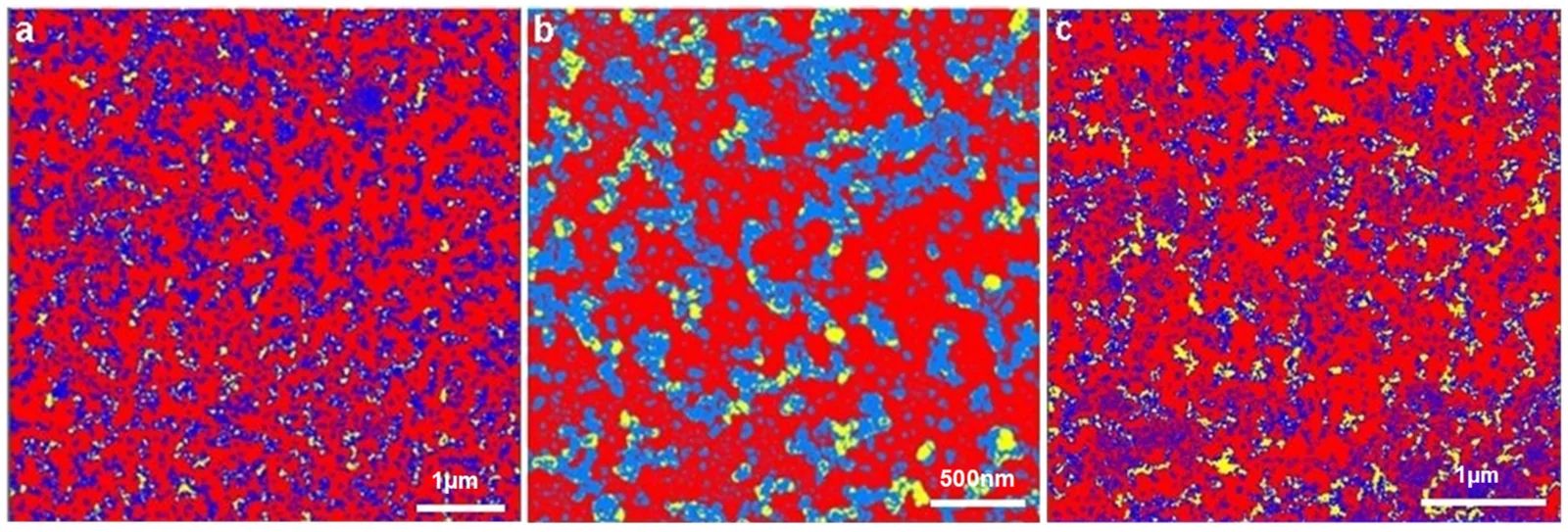
Analysis of Al_3_(Sc,Zr) and MgZn_2_ precipitates distribution in the microstructure, and volume fraction of precipitates in the alloy containing 0.1%Sc under thermomechanical treatment (the yellow regions are Al_3_(Sc,Zr) precipitates and the blue regions are MgZn_2_ precipitates) with: (**a**) MD30, (**b**) CD50, and (**c**) MD50 samples.

It can be said that, by using thermomechanical treatments, precipitate nucleation conditions are facilitated and more nucleation sites lead to a uniform distribution of precipitates throughout the structure. As the FESEM images (Figure 7 and Figure 8) show, Al_3_(Sc,Zr) and MgZn_2_ precipitates are uniformly distributed throughout the structure (instead of aggregation at the grain boundaries), which increases the strength of the structure and does not reduce ductility. These precipitates are dynamic precipitates created during thermomechanical treatments. In general, dynamic precipitation is related to the high density of point defects and the high recovery rate during rolling [61]. Precipitate distribution during thermomechanical and age hardening treatments creates effective sites for the trapping and accumulation of dislocations around precipitates. Therefore, during the tensile strain of aged specimens, the specimens will elongate before fracture (more ductility). Therefore, the combined effect of improved grain structure and precipitation hardening can be considered as the reason for increasing the strength of the alloy [62]. In the EDS analyses (Table 3, Table 4 and Table 5), elements of silicon and titanium are observed. Zhao et al. [63] reported that the presence of impurities, such as silicon, copper and titanium in the diffusion area around Al_3_(Sc,Zr) dispersoids increases the heterogeneous nucleation capacity of this phase. The volume fraction of precipitates during thermomechanical treatments (conventional dissolution and controlled quenching) is analyzed by MIP software and is shown in Figure 10. According to this analysis, the volume fractions of MgZn_2_ and Al_3_(Sc,Zr) precipitates in the sample MD30 were 33.91% and 6.66%, respectively, and in the sample CD30 were 32.58% and 4.94%, respectively, and in the sample MD50 were 30.43% and 8.21%, respectively.

The results of precipitate distribution analysis with MIP software performed on specimens undergoing T6 treatment were compared with the distribution of precipitates in specimens under thermomechanical treatment and are shown as a comparison in Table 6. It can be seen that, firstly, in thermomechanical treatments, the distribution of Al_3_(Sc,Zr) and MgZn_2_ precipitates has become more uniform than T6 heat treatment and also the volume fraction of precipitates has increased. The volume percentage of Al_3_(Sc,Zr) has increased from 0.38% in T6 treatment to 8.2% in sample MD50 and the volume percentage of MgZn_2_ from 7.67% to 33%. Secondly, with increasing the deformation, the volume fraction of precipitates in the microstructure has increased. As mentioned, the increase in precipitate nucleation sites and the less energy required for nucleation due to thermomechanical treatments is the reason for the increase in precipitate in the microstructure and their uniform distribution.

**Figure 11:**
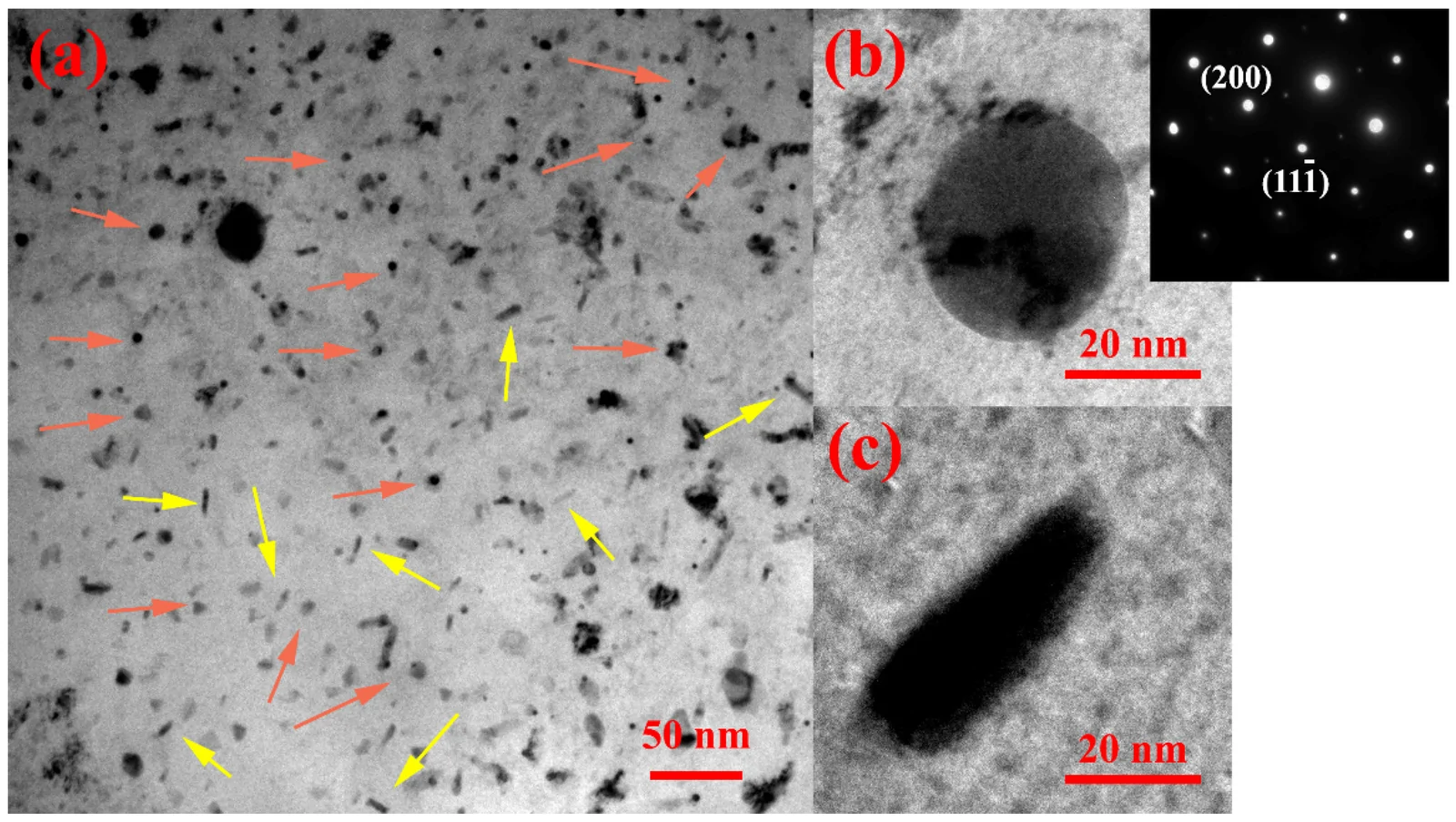
HR-TEM micrograph of the sampleMD50. (**a**) distribution of the spherical and rod shaped precipitates, (**b**) higher magnification of the spherical precipitate (**c**) higher magnification of the rod precipitate.

In order to more accurately study microstructure in the specimens under thermomechanical treatment, the specimen with the highest volume fraction of precipitates (sample MD50) was selected and its microstructure was investigated by TEM. The TEM micrograph of this specimen is shown in Figure 11. According to Figure 11, there are two types of precipitates in the microstructure with rod and spherical morphology. Some of the spherical particles are shown with 5–20 nm diameters (orange arrows) and rod-shaped particles with 20–40 nm diameters are shown with yellow arrows.

EDS analysis of particles with rod and spherical morphology is shown in Figure S3. According to the given EDS analysis, the spherical particles in the microstructure are of Al_3_(Sc,Zr) precipitates type and the rod particles are of MgZn_2_ type. The diffracted pattern in Figure 11 is related to an area of the Al matrix that is predominantly with spherical Al_3_(Sc,Zr) precipitates and is taken in the region axis (011). Superlattice diffractions such as (100) in the selected area diffraction patterns (SADP) confirm the existence of these precipitates [62].

### 3.4. Investigation of Mechanical Properties of the Alloys

The results of the tensile test of the specimens (UTS, QI, and elongation) and a comparison between the results of the present study with other studies are presented in Table 7. As can be seen in both T6 and thermomechanical treatments, the QI values in the controlled quenching methods are higher than that in the conventional dissolution methods. The QI obtained from thermomechanical treatment is more than the T6 heat treatment. The higher QI in thermomechanical treatments is due to the increase in the volume fraction of Al_3_(Sc,Zr) precipitates, their spherical morphology, and their uniform distribution throughout the structure, which leads to a simultaneous increase in tensile strength and elongation, resulting in increased QI. According to Table 7, with only 0.1 Sc, good elongation and strength and high QI were obtained. However, in studies reported with higher Sc values, this simultaneous increase in elongation and strength has not been achieved.

**Table 7:** Tensile properties of alloys and comparison between the results of the present study with other studies.

**Alloy**	$\sigma_{Y}\;(\mathrm{MPa})$	$\sigma_{\mathrm{UTS}}\;(\mathrm{MPa})$	%el	QI (MPa)	Ref.
MD30	514	560	16	745	This work
MD50	552	579	14	750.92	This work
CD30	599	611	10	750	This work
CD50	595	612	9.5	760	This work
Al-Zn-Mg-Cu-0.1Sc-0.1Zr (Age hardening with controlled quenching)	587	600.47	10	740	This work
Al-Zn-Mg-Cu-0.1Sc-0.1Zr (Conventional T6)	472.15	498.7	8	641	This work
Al-5.41Zn-1.98Mg-0.33Cu-0.25Sc-0.1Zr (T6)	524	555	12.3	718	[64]
Al-Zn-Mg-Mn-0.1Sc (T6)	400	-	13	567	[65]
7075-0.25Sc-0.15Zr (T6)	626	568	10	770	[27]
Al-Zn-Mg-Cu-0.2Sc-0.4Zr (T6)	667	627	10	818	[39]
7075 (T6)	371	-	12	531	[66]

The results show that the tensile strength of the alloy containing 0.1%Sc with thermomechanical and ageing treatments has increased by 25% compared to the alloy under T6 treatment, so that the tensile strength of the alloy has increased from 490 MPa to 620 MPa. This is while the rate of elongation has increased. However, ductility is expected to decrease with increasing strength. While with thermomechanical treatments, in addition to increasing strength, ductility has also increased compared to T6 treatments. It can also be seen that, with controlled quenching thermomechanical treatments, the ductility increased to 16% while the tensile strength decreased by only 10% compared to thermomechanical treatments with conventional dissolution. In fact, there is a 10% decrease in strength and 50% increase in ductility in thermomechanical treatments with modified quenching compared to thermomechanical treatments with conventional dissolution. This change in tensile strength and ductility relative to the alloy with T6 treatment is 20% and 70%, respectively. In the thermomechanical treatment, after the dissolution, the rolling was performed at 100 °C temperature in successive passes. Since this temperature is the recovery temperature of the alloy, so after each rolling pass, remaining at 100 °C helps to recover the effects of dislocations and increases ductility, and on the other hand, the energy required for precipitate nucleation is provided and the number of precipitate nucleation sites in the matrix increases. FESEM images show that a large number of precipitates are uniformly distributed in the matrix, so the main reason for increasing the strength of the specimens is strengthening with precipitates. These precipitates prevent dislocation movements. In fact, the size and distribution of precipitates and discontinuities of precipitates have a great effect on improving the mechanical properties of Al-Zn-Mg-Cu alloy [67]. Simultaneous acquisition of strength and ductility in Al alloys is difficult [31]. Improved ductility in specimens can be due to the reduction of dislocations during age hardening treatments, and the presence of nano-sized dispersoids in the aged specimens. Because the density of precipitates is high, more dislocations accumulate around the precipitates, causing a long-term tensile strain and increasing ductility [62].

Precipitate distribution affects hardness, tensile strength and the failure mechanism. When the precipitates are small and well distributed, the strength and hardness are greater than when the precipitates are large. Therefore, in order to achieve high hardness and strength, heat treatment should be done in such a way that fine precipitates are created with uniform distribution in the structure [68,69,70]. In the present study, Al_3_(Sc,Zr) precipitates are small with a uniform distribution among MgZn_2_ precipitates. The morphology and distribution of these phases play an essential role in the performance of the alloy [34]. Small spherical homogeneous dispersoids improve plasticity. These particles and phases participate in strengthening processes [35]. Therefore, the interaction between dislocations and precipitates increases the mechanical properties obtained in thermomechanical treatments [55]. It has been shown that thermomechanical treatments greatly affect the mechanical properties and microstructure of Al alloys [36]. Therefore, by performing thermomechanical treatments, tensile strength and elongation can be increased simultaneously [38].

### 3.5. Investigation of Fracture Cross-Section

**Figure 12:**
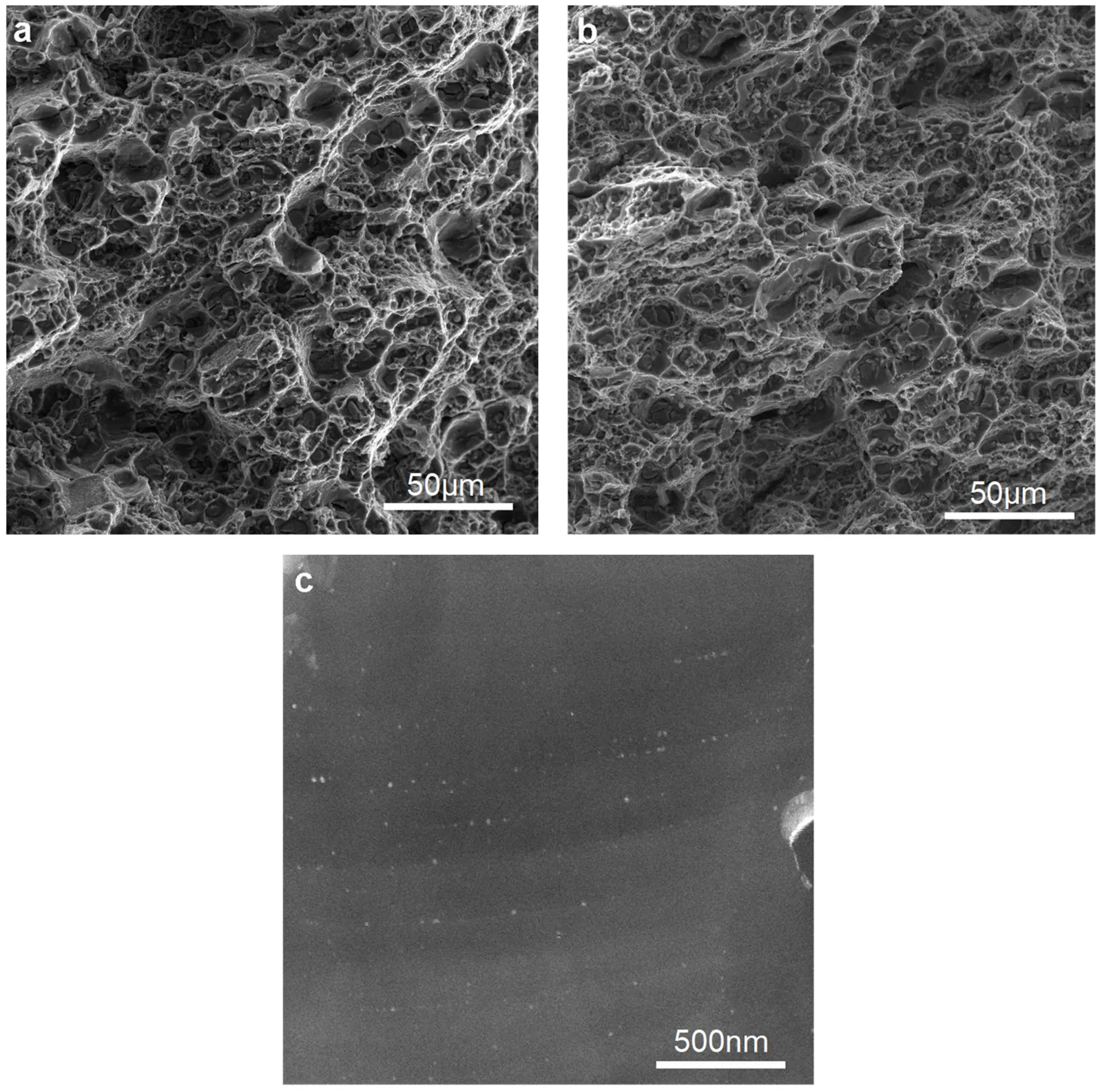
Dimples on the fracture surface in the specimen under thermomechanical treatment with controlled quenching and (**a**) 30% rolling, and (**b**) 50% rolling; (**c**) nano-precipitates at the end of dimples.

Fracture is often the end point of deformation processes. Therefore, fracture surfaces can indicate the deformation processes to which matter has been exposed [39]. The mechanical properties of materials directly depend on their fracture mechanism, especially on the processes of crack growth and microscopic fracture. Therefore, qualitative fracture study has a fundamental role in materials research. FESEM has been used for this purpose. FESEM images of the fracture surfaces of the specimens under thermomechanical treatment, on which the tensile test was performed, are shown in Figure 12.

The volume fraction of precipitates at the fracture surface and the average size and the percentage of dimples at the fracture surface, which determine the type of fracture, are analyzed using MIP software and their comparison results are presented in Table 8.

At the fracture surface of the samples, a large number of fine dimples are observed. The type of fracture indicates the inter-connection of micro-cavities whose formation, growth and their inter-connection are the dominant phenomena in the fracture mechanism [40]. The size of dimples in the specimen with less deformation (30%) is smaller than the specimen with more deformation. Fracture surfaces have dimples that are characteristic of ductile fracture. The results of MIP analysis also show that, in thermomechanical treatments with controlled quenching, there are more uniform dimples in terms of size. According to Table 8, with increasing the deformation rate, the size of the dimples has become larger, which can be due to more deformation during thermomechanical treatments. However, in general, it is observed that the percentage of dimples in the fracture surface of samples under thermomechanical treatment is higher than the sample under T6 with conventional dissolution and there is more ductility in samples under thermomechanical treatment than conventional T6 heat treatment and therefore a more ductile fracture. Additionally, in samples undergoing thermomechanical treatment with controlled quenching, the size of the dimples is smaller than the samples under thermomechanical treatment with conventional dissolution. Many microcracks start from these large dimples. The formation of fine Al_3_(Sc,Zr) precipitates in aged alloys can create suitable areas for crack nucleation. The fracture surface of the specimens shows that the predominant fracture phenomenon is a semi-ductile fracture and some brittle fracture is also seen in the fracture surfaces.

As can be seen in the fracture surface images of the specimens, a large number of nanometer-sized Al_3_(Sc,Zr) dispersoids are present in the fracture surface. The presence of these nano precipitates at the fracture surface may indicate that the probable mechanism of fracture in these specimens is the Orowan mechanism. Other research has shown that the mechanism of Al_3_Sc nano-sized precipitate strengthening is the Orowan mechanism (dislocation bypassing behavior) [71,72]. The joining of dimples together during tensile deformation causes the formation of large cracks and eventually the fracture. As the strain increases, the new cavities become larger and the failure process progresses as these cavities join together. Joining cavities is an internal necking mechanism that often occurs at low to moderate stress levels [73]. In the specimen with a higher percentage of deformation, the shape and size of the dimples become larger and a more compact dimple structure is created at the fracture surface. Nucleation and joining of cavities and dimples may lead to the growth of cracks along the grain boundaries.

## 4. Conclusions

Thermomechanical treatments consisted of three stages of dissolution, a multi-pass rolling process at 100 °C recovery temperature and hardening at a temperature of 120 °C for 12 h. The age hardening temperature in this treatment for specimens with 50% and 30% deformation was obtained at 120 °C and 130 °C, respectively. Tensile and yield strength increased 110% in specimens with thermomechanical treatment compared to annealed specimens, while the elongation has not changed much. In the T6 age hardening treatment, tensile/yield strength and elongation compared to the annealed specimen increased by 50% and decreased by 40%, respectively. While tensile/yield strength and elongation in thermomechanical treatment increased by 25% and 50% compared to T6 treatment, respectively. A simultaneous increase in tensile/yield strength and elongation in these specimens is due to the uniform distribution of Al_3_(Sc,Zr) nano-dispersoids throughout the microstructure and among MgZn_2_ dispersoids. Investigation of the fracture surfaces showed that Al_3_(Sc,Zr) nano-dispersoids were distributed at the fracture surface and the fracture surface consisted of small dimples indicating the semi-ductile fracture of the alloy, and the possible fracture mechanism is the Orowan mechanism.

## Figures and Tables

**Table 1:** Chemical composition of Al-Zn-Mg-Cu-0.1%Zr-0.1%Sc (wt.%).

Al	Zn	Mg	Cu	Ti	Mn	Cr	Ni	Fe	Be	Pb	Sn	Zr	Si	Sc
balanced	6.14	3.32	1.20	0.07	0.093	0.17	0.009	0.24	0.0007	0.0025	0.0093	0.1	0.153	0.1

**Table 2:** Specifications of Al-Zn-Mg-Cu-0.1%Zr-0.1%Sc alloys and the performed thermomechanical treatments.

Alloy Code	Thermomechanical Treatment
CD30	Homogenizing + Rolling + Secondary annealing + Dissolution + 30% rolling at 100 °C + Ageing
CD50	Homogenizing + Rolling + Secondary annealing + Dissolution + 50% rolling at 100 °C + Ageing
MD30	Homogenizing + Rolling + Secondary annealing + Modified dissolution + 30% rolling at 100 °C + Ageing
MD50	Homogenizing + Rolling + Secondary annealing + Modified dissolution + 50% rolling at 100 °C + Ageing

**Table 3:** EDS analysis of points A and B in Figure 7.

Phase	Chemical Composition (%at.)					
	Al	Sc	Zr	Mg	Zn	Ti
A	93.54	-	-	1.55	3.12	-
B	95.91	0.38	1.52	-	1.96	0.23

**Table 4:** EDS analysis of points A and B in Figure 8.

Phase	Chemical Composition (%at.)						
	Al	Sc	Zr	Mg	Zn	Ti	Si
A	95.65	1.48	2.59	-	1.49	0.1	0.18
B	94.86	-	-	3.22	1.92	-	-

**Table 5:** EDS analysis of points C and D in Figure 9.

Phase	Chemical Composition (%at.)					
	Al	Sc	Zr	Mg	Zn	Si
C	95.29	-	-	2.92	1.49	0.3
D	88.97	2.82	6.09	2.48	-	-

**Table 6:** Comparison of volume fraction of Al_3_(Sc,Zr) and MgZn_2_ precipitates in the alloys containing 0.1% Sc in specimens under different treatment conditions, using MIP software analysis.

Treatment	Volume Fraction of MgZn_2_ (%)	Volume Fraction of Al_3_(Sc,Zr) (%)
Age hardening with controlled quenching	17.91	1.96
Age hardening with conventional dissolution	2.76	0.38
MD30	33.91	6.66
MD50	30.43	8.2
CD30	32.58	4.94

**Table 8:** Comparative results of analysis of dimples on the fracture surface using MIP software.

Treatment	Volume Fraction of Dimples at the Fracture Surface (%)	Mean Size of Dimples (μm)	Volume Fraction of MgZn_2_ at the Fracture Surface (%)	Volume Fraction of Al_3_(Sc,Zr) at the Fracture Surface (%)
Age hardening with controlled quenching	38.5	55.88	17.65	2.69
Age hardening with conventional dissolution	32.87	92.66	7.67	0.38
MD30	38.23	81	35.3	0.14
MD50	33.63	140	32.58	4.94
CD30	44.33	101	30.43	8.21

## Data Availability

All the data are available within the manuscript.

## Supplementary Materials

The following supporting information can be downloaded at: https://www.mdpi.com/article/10.3390/ma15020589/s1, Figure S1: Metallographic image of alloy containing 0.1% Sc under thermomechanical treatment with conventional dissolution, after dissolution, and 50% deformation.; Figure S2: Metallographic image of alloy containing 0.1% Sc under thermomechanical treatment with controlled quenching: (**a**) after dissolution and 30% deformation, (**b**) ageing at 130 °C, (**c**) after dissolution and 50% deformation, and (**d**) ageing at 120 °C.; Figure S3: EDS analysis of dispersoids in the TEM micrograph given in Figure 12: (**a**) spherical particles, and (**b**) rod particles.
