# Correction: Beigi Kheradmand et al. Effect of Thermomechanical Treatment of Al-Zn-Mg-Cu with Minor Amount of Sc and Zr on the Mechanical Properties. *Materials* 2022, *15*, 589

**DOI:** 10.3390/ma19153241

**Published:** 2026-07-31

**Authors:** Azam Beigi Kheradmand, Shamseddin Mirdamadi, Zahra Lalegani, Bejan Hamawandi

**Affiliations:** 1 Department of Mechanical Engineering, Shahrekord Branch, Islamic Azad University, Shahrekord 88137-33395, Iran; 2 Department of Materials Engineering, Science and Research Branch, Islamic Azad University, Tehran 82448-65179, Iran; mirdamadi@iust.ac.ir; 3 Young Researchers and Elite Club, Shahrekord Branch, Islamic Azad University, Shahrekord 88137-33395, Iran; z.lalegani@ut.ac.ir; 4 Department of Applied Physics, KTH Royal Institute of Technology, SE-10691 Stockholm, Sweden

In the original publication [1], the citation referring to Reference 73 in the manuscript has been verified by academic editors to be irrelevant. Concerns were raised on this matter, and the following reference was removed from the reference list:

73. Liu, J.; Mao, S.; Song, S.; Huang, L.; Belfiore, L.A.; Tang, J. Towards applicable photoacoustic micro-fluidic pumps: Tunable excitation wavelength and improved stability by fabrication of Ag-Au alloying nanoparticles. *J. Alloys Compd.* **2021**, *884*, 161091.

With this correction, the order of some references has been adjusted accordingly. The authors state that the scientific conclusions are unaffected. This correction was approved by the Academic Editor. The original publication has also been updated.
