# Supplementary material for: Effect of Thermomechanical Treatment of Al-Zn-Mg-Cu with Minor Amount of Sc and Zr on the Mechanical Properties
Source: Materials (Basel). 2022 Jan 13;15(2):589. doi: 10.3390/ma15020589 (PMC8777705; doi:10.3390/ma15020589)
Supplement: Supplementary file 1 [file materials-15-00589-s001.zip › materials-1470863-supplementary.pdf]

materials

Supplementary Material

Effect of Thermomechanical Treatment of Al-Zn-Mg-Cu with Minor Amount of Sc and Zr on the Mechanical Properties

Azam Beigi Kheradmand[1,*], Shamseddin Mirdamadi[2], Zahra Lalegani[3] and Bejan Hamawandi[4,*]

1 Department of Mechanical Engineering, Shahrekord Branch, Islamic Azad University, 88137-33395 Shahrekord, Iran
2 Department of Materials Engineering, Science and Research Branch, Islamic Azad University, 82448-65179 Tehran, Iran; mirdamadi@iust.ac.ir
3 Young Researchers and Elite Club, Shahrekord Branch, Islamic Azad University, 88137-33395 Shahrekord, Iran;
z.lalegani@ut.ac.ir
4 Department of Applied Physics, KTH Royal Institute of Technology, SE-10691 Stockholm, Sweden
* Correspondence: kheradmand@iaushk.ac.ir (A.B.K.); bejan@kth.se (B.H.)

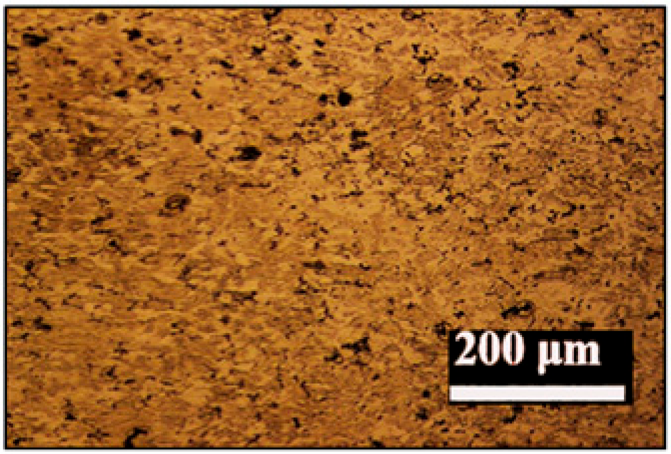

Figure S1. Metallographic image of alloy containing 0.1% Sc under thermomechanical treatment with conventional dissolution, after dissolution, and 50% deformation.

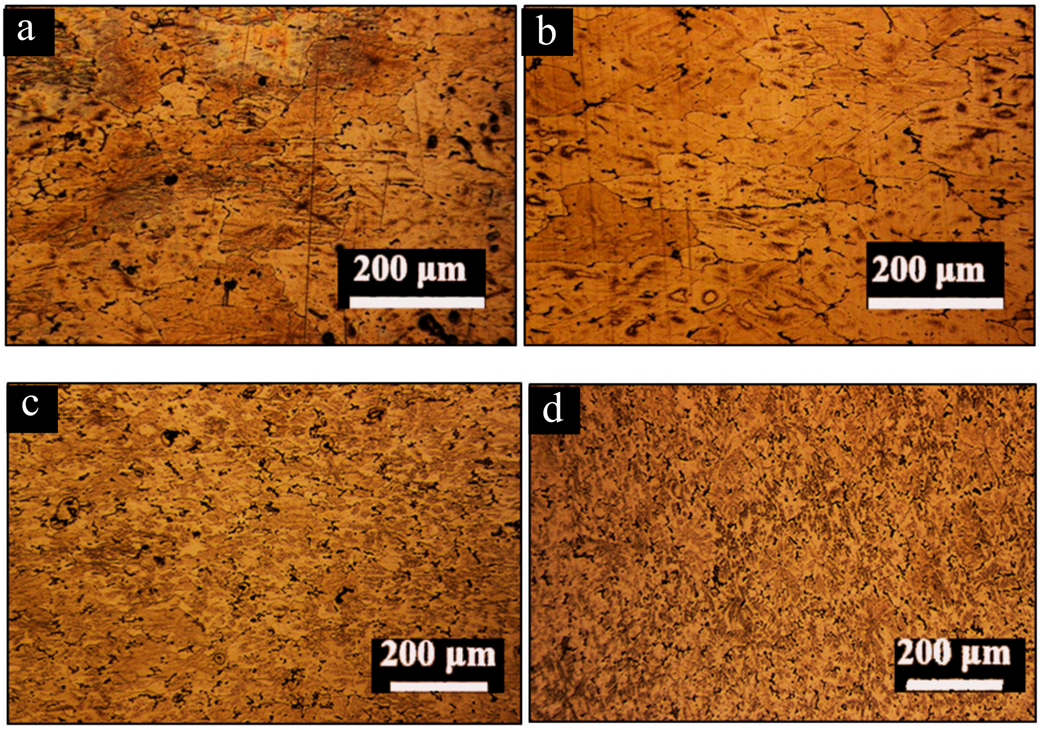

Figure S2. Metallographic image of alloy containing 0.1% Sc under thermomechanical treatment with controlled quenching: (**a**) after dissolution and 30% deformation, (**b**) ageing at 130°C, (**c**) after dissolution and 50% deformation, and (**d**) ageing at 120°C.

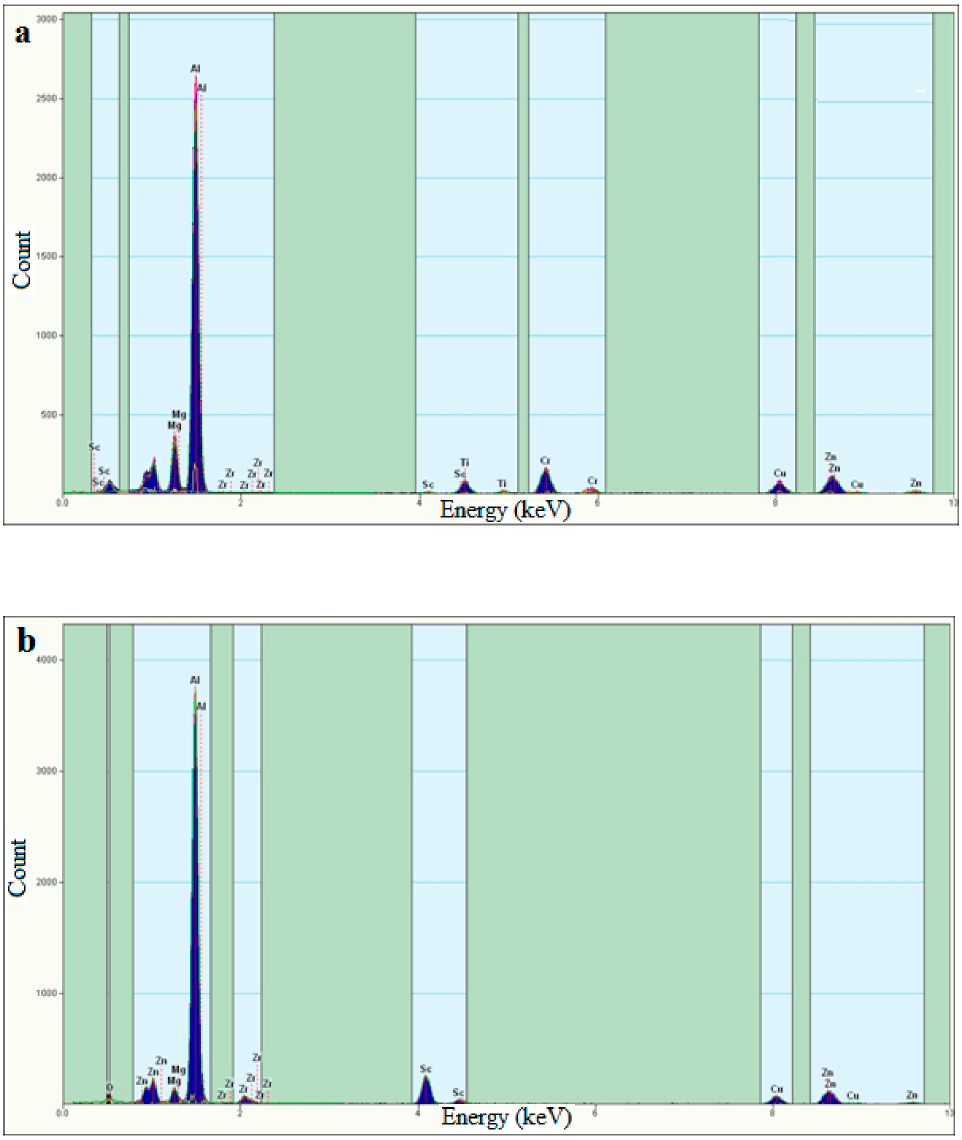

Figure S3. EDS analysis of dispersoids in the TEM micrograph given in figure 12: (**a**) spherical particles, and (**b**) rod particles.
